# Revolutionizing Pediatric Myopia Care: A Machine Learning Approach for Rapid and Accurate Pre-Clinical Screening

**DOI:** 10.3390/jcm15082834

**Published:** 2026-04-08

**Authors:** Siqi Zhang, Qi Zhao

**Affiliations:** Department of Ophthalmology, The Second Affiliated Hospital of Dalian Medical University, Zhongshan Road 467, Shahekou District, Dalian 116027, China; dr.zhangsiqi@outlook.com

**Keywords:** artificial intelligence, myopia diagnosis, children and adolescents, machine learning

## Abstract

**Background/Objective:** Myopia has become a prominent public health issue in China, significantly impacting the visual health of children and adolescents. The condition is characterized by a high incidence rate, increasing prevalence, and a trend toward earlier onset, highlighting the critical need for early and accurate diagnosis. Current clinical diagnostic methods primarily depend on subjective evaluations by optometrists and the use of isolated parameters, leading to inefficiencies and inconsistent outcomes. Moreover, there remains a lack of diagnostic tools that can effectively integrate multi-parameter analysis while ensuring robust data privacy protection. This study aims to develop an artificial intelligence (AI) diagnostic model that achieves objective, accurate, and safe diagnosis of myopia in children without cycloplegia through multi-parameter fusion and to enable local deployment. The proposed model is intended to be a reliable tool for clinical applications and large-scale screening projects, while ensuring strong protection of patient privacy. **Methods:** We built a transparent, rule-driven AI framework using clinical guidelines. Key ocular parameters—visual acuity, spherical equivalent, axial length, corneal curvature, and axial ratio—were encoded as logical rules in Python and incorporated via instruction fine-tuning. The model was trained and validated on retrospective clinical data (70% training, 15% validation, 15% test) using five algorithms: gradient boosting, logistic regression, random forest, SVM, and XGBoost. Performance was evaluated using accuracy, precision, recall, F1 score, and mean AUC across classes. **Results:** The model classifies refractive status into five categories: hyperopia, pre-myopia, mild, moderate, and high myopia. All five different algorithms demonstrated excellent diagnostic and classification performance. Gradient boosting achieved the best overall performance, with an accuracy of 98.67%, an F1 score of 98.67%, and a mean AUC of 0.957—outperforming all other models. **Conclusions:** This study successfully developed an artificial intelligence-based myopia diagnosis system for children under non-dilated pupil conditions. The system is interpretable and privacy-preserving, and has excellent diagnostic and classification performance, making it suitable for clinical decision support and large-scale screening applications. It has great potential to promote the development of early intervention, precision prevention, and control strategies for childhood myopia.

## 1. Introduction

Myopia among children and adolescents has emerged as a significant public health challenge in China. According to the World Health Organization (WHO), the global prevalence of myopia exceeds 33%, while in China, the overall myopia rate among children and adolescents reaches 52.7%, rising to 80.5% among high school students. Furthermore, the condition is increasingly affecting younger age groups and progressing toward higher degrees, indicating a concerning trend of earlier onset and greater severity [1,2,3,4]. Myopia not only impairs visual function but is also associated with adverse psychological outcomes and imposes a substantial economic burden on individuals and society. From a pathological standpoint, axial myopia—accounting for over 85% of cases—poses greater risks, as each millimeter increase in axial length corresponds to an approximate myopic shift of 3.00 diopters [5,6]. Moreover, accelerated axial elongation significantly elevates the risk of sight-threatening fundus complications. When axial length exceeds 26 mm, the likelihood of pathological changes such as tessellated fundus, retinal degeneration, and other posterior segment abnormalities increases markedly [7]. Therefore, early and accurate diagnosis is critical for effective intervention and progression control. In recent years, myopia prevention and control have been elevated to a national strategic priority, with the implementation of comprehensive public health initiatives. Nevertheless, disparities in clinical resource distribution and an incomplete screening infrastructure continue to limit the effectiveness of current efforts. Hence, the development of a scientifically grounded, efficient screening and intervention system is urgently needed to safeguard the visual health of the younger population.

Myopia diagnosis continues to face significant challenges in clinical practice. Current approaches rely heavily on the subjective judgment and expertise of optometrists and refractive specialists, with spherical equivalent refractive error serving as the primary—often sole—diagnostic criterion. This reliance renders assessments vulnerable to variability due to patient cooperation, accommodation, and environmental factors during examination. Furthermore, myopia progression is a multifactorial process involving dynamic changes in axial length, corneal curvature, and accommodative function. Relying exclusively on refractive error limits the ability to comprehensively evaluate both the current myopic status and future progression risk. In primary healthcare settings and large-scale school-based screening programs, the absence of standardized, objective diagnostic tools leads to suboptimal screening efficiency and poor inter-examiner consistency. Consequently, there is an urgent need to develop and implement a multimodal, parameter-integrated assessment model to enable precise, reliable, and standardized diagnosis of myopia.

Artificial intelligence has achieved notable advancements in ophthalmology, with applications in fundus image analysis for the detection of age-related macular degeneration, vascular diseases, diabetic retinopathy, and pathological myopia. Leveraging deep learning algorithms, AI systems enable automated lesion detection, quantitative assessment, and computer-aided diagnosis with high precision [8,9,10]. However, its application in pediatric myopia diagnosis remains limited, especially due to the absence of comprehensive artificial intelligence models that integrate multiple biometric parameters—such as axial length, corneal curvature, and refractive error—into a unified analytical framework. Most current studies are confined to single-parameter analyses, which fail to capture the multifactorial nature of myopia progression and thus limit risk stratification accuracy. Research on multi-parameter AI-driven approaches tailored specifically for children is still insufficient. Therefore, there is an urgent need to integrate longitudinal clinical data with advanced machine learning techniques to develop robust, standardized, and clinically applicable intelligent diagnostic systems for early and comprehensive assessment of myopia in pediatric populations.

**Figure 1:**
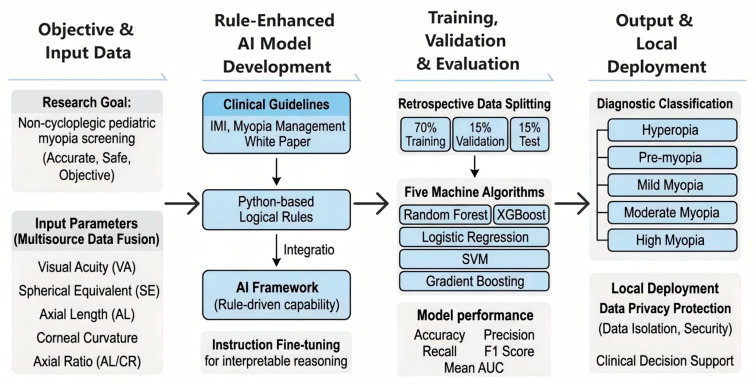
Development and Evaluation of a Rule-Enhanced Al Model for Non-Cycloplegic Pediatric Myopia Diagnosis.

To address the aforementioned challenges, this study proposes a “rule-enhanced artificial intelligence” framework (Figure 1). By integrating established myopia definitions and diagnostic criteria from national and international clinical guidelines with the deep semantic understanding and logical reasoning capabilities of large language models, and by synthesizing multimodal clinical parameters—including visual acuity, refractive error, axial length, corneal curvature, and the axial length-to-corneal curvature ratio (ACR)—we aim to develop an AI-driven diagnostic model that combines clinical validity with advanced cognitive reasoning. This model is expected to improve the efficiency and consistency of myopia diagnosis in clinical practice and serve as a reliable tool for large-scale screening in primary care and school-based settings, thereby supporting early intervention and prevention strategies. Furthermore, through a localized deployment architecture, all patient medical data are processed within a secure, closed environment, ensuring full compliance with privacy protection standards and safeguarding data security.

## 2. Materials and Methods

### 2.1. Ethical Approval

The research protocol of this study underwent scientific review and was formally approved by the Research Department of the Second Affiliated Hospital of Dalian Medical University. It was subsequently reviewed and approved by the Institutional Ethics Committee of the same institution, in accordance with ethical standards for medical research involving human subjects (Ethical Approval Number: KY2025-182-01).

### 2.2. Study Design and Data Sources

A retrospective observational design was used to include patients who visited the Pediatric Ophthalmology Strabismus and Refractive Error Clinic at the Second Affiliated Hospital of Dalian Medical University between January 2025 and December 2025, and who met the pre-defined inclusion and exclusion criteria. The detailed eligibility criteria are as follows:

Inclusion Criteria:

(1) Provision of written informed consent by the participant and/or their parent(s)/legal guardian(s);
(2) Age between 6 and 12 years, inclusive;
(3) Clinical diagnosis of pre-myopia or myopia established by the same experienced senior ophthalmologist specializing in refractive disorders;
(4) Generally healthy status with no significant ocular comorbidities or systemic chronic diseases that would impair participation in the study.
(5) No exclusion criteria were applied.

Exclusion Criteria:

(1) Inability to cooperate with required ophthalmic examinations;
(2) Presence of ocular conditions including elevated intraocular pressure, corneal pathology, active ocular inflammation, cataract, glaucoma, fundus abnormalities, or significant opacities of the refractive media;
(3) Presence of amblyopia, strabismus, or nystagmus that precludes stable visual fixation;
(4) Known systemic diseases that may affect ocular or general health;
(5) Prior receipt of any intervention potentially associated with visual improvement;
(6) Participation in another clinical study within the preceding three months.

This study enrolled a total of 1436 children and adolescents aged 6 to 12 years. The overall dataset comprised comprehensive and valid data records across multiple domains, including visual acuity, refractive parameters, and ocular biometric measurements, amounting to 12,008 individual data points, thereby providing robust support for model training and validation. Key parameters were collected using standardized methodologies: visual acuity was measured using the LogMAR chart; spherical and cylindrical refractive errors were obtained with the Topcon KR-8900 automated refractometer (Topcon Healthcare, Oakland, NJ, USA); axial length was measured using the IOLMaster 700 biometer (Carl Zeiss Meditec AG, Jena, Germany), and corneal curvature (CR) was captured using the Pentacam Scheimpflug imaging system (OCULUS Optikgeräte GmbH, Wetzlar, Germany). Furthermore, the axial length-to-corneal radius ratio (AL/CR) was calculated as an index reflecting myopic progression. These parameters are established clinical indicators for assessing the onset and development of myopia. All examinations were performed following standardized operating protocols to ensure data consistency, accuracy, and reliability.

### 2.3. Data Preprocessing

During the data preprocessing phase, this study strictly adhered to established machine learning protocols to improve data quality and enhance model generalization performance. Z-score normalization was performed using StandardScaler: the mean and standard deviation were computed from the training set, and these parameters were subsequently applied to the test set to maintain methodological consistency. Missing values in numerical variables were imputed using the median to minimize the influence of outliers on the imputation process. Outlier detection and handling involved a multi-stage validation procedure: initially, clinically plausible thresholds were defined based on medical knowledge—for instance, an axial length exceeding 30 mm was considered implausible and flagged as a potential outlier; subsequently, all flagged cases were independently reviewed by at least two ophthalmologists to determine whether they resulted from measurement errors or represented genuine pathological conditions. Data points confirmed as erroneous were excluded from further analysis to ensure dataset reliability.

Additionally, gender data were uniformly encoded using the convention 0 = male and 1 = female, ensuring compatibility across both numerical and string data formats. For age processing, values in the format “6/0” were parsed by extracting the numerator, and the resulting numerical value was used directly in analysis. To ensure patient privacy and data integrity, a unique 16-character alphanumeric identifier was generated for each individual using the SHA256 cryptographic hash function, formatted as A1B2-C3D4-E5F6-G7H8.

### 2.4. The Construction of the “Rule-Enhanced AI” Model

In the context of this study’s rule-enhanced framework, “instruction fine-tuning” does not refer to traditional large language model tuning, but rather to the process of encoding authoritative clinical guidelines into logical constraints (as shown in Algorithm 1). These logical outputs were provided as explicit “instructions” (weighted categorical features) to guide the training of the traditional machine learning classifiers (RF, XGBoost, etc.). This ensures the algorithms prioritize established clinical boundaries over pure statistical correlations.
**Algorithm 1:** Rule-Enhanced Clinical Logic for Refractive Status and Risk StratificationInput: Age(years),SE(Spherical Equivalent, Diopters),AL(Axial Length, mm),CR(Corneal Radius, mm)Output: Refractive_Category(Class label),Progression_Risk_Flag(Binary)1: Initialize Refractive_Category = “Unknown”2: Initialize Progression_Risk_Flag = 03:4: /* Step 1: Calculate biometric derived features */5: Compute AL_CR_Ratio = AL / CR6: Extract Hyperopia_Reserve_Upper (from Table 1 based on Age)7: Extract AL_Max (from Table 2 based on Age)8:9: /* Step 2: Refractive Status Classification (Non-cycloplegic rules) */10: IF SE > Hyperopia_Reserve_Upper THEN11:    Refractive_Category = “Hyperopia”12: ELSE IF SE > 0.75 THEN13:    Refractive_Category = “Pre-myopia” // Borderline high hyperopia not in reserve14: ELSE IF SE >= −0.50 AND SE <= 0.75 THEN15:    Refractive_Category = “Pre-myopia”16: ELSE IF SE >= −3.00 AND SE < −0.50 THEN 17:    Refractive_Category = “Low myopia” // Note: Clinical implementation uses SE <= −0.75 D for non-cycloplegic18: ELSE IF SE >= −6.00 AND SE < −3.00 THEN19:    Refractive_Category = “Moderate myopia”20: ELSE IF SE < −6.00 THEN21:    Refractive_Category = “High myopia”22: END IF23:24: /* Step 3: Structural Risk Flagging (Addressing non-cycloplegic accommodation error) */25: IF AL_CR_Ratio >= 3.00 OR AL > AL_Max THEN26:    Progression_Risk_Flag = 1  // Indicates high risk of structural axial elongation27: ELSE28:    Progression_Risk_Flag = 029: END IF30:31: RETURN Refractive_Category, Progression_Risk_Flag

Furthermore, the “self-healing mechanism” refers to our robust, automated data preprocessing pipeline described in Section 2.3. During model inference, if missing or biologically implausible values (e.g., AL > 30 mm) are detected, the system automatically triggers a “self-healing” protocol: it applies median imputation based on age and gender strata for minor deviations, or flags extreme anomalies for manual review, thereby preventing model failure and ensuring stable clinical predictions.

#### 2.4.1. Core Reference Indicators Embedded in the Clinical Guidance Framework

This study developed a comprehensive set of diagnostic rules for pediatric myopia using the Python 3.12 programming language. The rule development process strictly adhered to established domestic and international clinical guidelines for myopia diagnosis and management, incorporating key ophthalmic parameters including visual acuity (VA), spherical equivalent (SE), axial length (AL), corneal curvature (K), corneal curvature radius (CR), and axial ratio (AL/CR). By integrating logical inference rules with machine learning algorithms, the framework enables automated detection and risk stratification of myopia. The specific implementation is outlined below:

This study leverages established definitions and threshold criteria for myopia-related risk factors—such as axial length (AL) and axial ratio (AL/CR)—from authoritative domestic and international clinical guidelines, including the “International Myopia Institute (IMI) White Paper on Myopia Control Research (2023)” [11], the “Myopia Management White Paper 2025” [12], the “Expert Consensus on Reference Intervals for Ocular Hyperopia Reserve, Axial Length, Corneal Curvature, and Related Genetic Factors in Chinese School-Aged Children (2022)” [13], the “Expert Consensus on the Application of Axial Length in Myopia Control and Management (2023)” [14], and the “Expert Consensus on Phased Public Health Strategies for Myopia Prevention and Control in Children and Adolescents (2022)” [15]. These guidelines’ qualitative diagnostic criteria and quantitative cut-off values were systematically translated into executable computational logic rules. A preliminary decision tree model was developed based on multiple ophthalmic parameters—including spherical equivalent (SE), axial length (AL), corneal curvature (CR), and derived axial ratio (AL/CR)—to enable automated diagnosis and severity stratification of myopia, categorizing individuals into clinically meaningful groups: hyperopia, emmetropia/pre-myopia, low myopia, moderate myopia, and high myopia.

The operational definitions of the key biological parameters are specified as follows: Visual acuity was used as a supplementary clinical criterion for distinguishing abnormal from normal cases; for children aged 6 to 12 years, the lower threshold for normal visual acuity was defined as 0.7 [11]. The reference values for hyperopia reserve are provided in Table 1 [12,13]; axial length reference values in Table 2 [14]; and corneal curvature radius reference values in Table 3 [13]. For the axial ratio (AL/CRC), prior studies have identified an optimal diagnostic threshold of 3.00, which corresponds to the maximum Youden index. This value is highly consistent with the cutoff of 2.99 reported by He X et al. and aligns exactly with the findings of Goss DA et al. Therefore, to ensure computational simplicity and consistency in implementation, the axial ratio threshold was set at 3.00 in this study [16,17].

**Table 1:** Reference range for farsighted reserve in school-aged children aged 6–15.

Age (Years)	Mean (D)	Reference Interval (D)
6	+1.38	+0.38~+3.63
7	+1.38	+0.38~+3.63
8	+1.25	+0.38~+3.38
9	+0.88	+0.13~+3.13
10	+0.75	−0.13~+2.88
11	+0.63	−0.38~+2.88
12	+0.50	−0.38~+2.50

**Table 2:** Reference values for the axial length of the eyes of children and adolescents.

Age	Axial-Min (mm)	Axial-Max (mm)	Axial-Mean (mm)
6	20.93	23.98	22.46
7	21.07	24.04	22.68
8	21.3	24.27	22.9
9	21.45	24.46	23.05
10	21.6	24.67	23.22
11	21.71	24.8	23.38
12	21.79	24.84	23.52

**Table 3:** Reference values for corneal curvature radius of children and adolescents.

Age	Corneal-Min (mm)	Corneal-Max (mm)	Corneal-Mean (mm)
6	7.93	8.45	8.19
7	7.09	8.7	7.895
8	7.42	8.41	7.915
9	7.41	8.43	7.92
10	7.41	8.43	7.92
11	7.42	8.41	7.915
12	7.39	8.43	7.91

All clinical variables were entered as structured numerical features. The refractive category was defined primarily by spherical equivalent derived from autorefractor measurements according to predefined thresholds. Axial length and corneal curvature were included as auxiliary predictive variables, particularly for borderline cases, rather than as direct diagnostic criteria. These variables were jointly input into the machine learning models to enable integrated prediction based on both refractive and biometric information.

#### 2.4.2. Diagnostic Logic Description

Refractive Threshold: According to international diagnostic criteria for myopia, the condition is defined as an equivalent spherical refractive power (SE) of less than −0.50 D following cycloplegic mydriasis. However, a major challenge in large-scale pediatric screening is the impracticality of routine cycloplegia. Consequently, all data fed into the AI model were collected under non-cycloplegic conditions. To compensate for the potential overestimation of myopia due to accommodative spasm, our framework implements two specific adjustments. First, we adopted a stricter spherical equivalent threshold (SE ≤ −0.75 D instead of the standard −0.50 D) [11]. Second, the model relies heavily on structural ocular biometrics—namely Axial Length (AL), Corneal Radius (CR), and the AL/CR ratio. Because these anatomical parameters are completely invariant to accommodative status, their integration allows the machine learning models to effectively differentiate between true axial elongation and transient pseudomyopia, ensuring high diagnostic accuracy even without cycloplegia.

The diagnostic classification criteria for different refractive states are presented in Table 4.

Factors requiring special consideration include axial eye length deviation, corneal curvature deviation, and pupillary dilation status. The degree of deviation for each parameter should be expressed as a percentage difference relative to the age-matched reference mean, whereas pupillary dilation status influences the choice of refractive threshold.

#### 2.4.3. Algorithmic Pseudocode for Rule-Based Classification

To ensure transparency and reproducibility, the clinical guidelines detailed in Table 1, Table 2, Table 3 and Table 4 were synthesized into a unified logical framework. The following pseudocode illustrates the core decision-making logic used to generate baseline risk stratifications, as detailed in Algorithm 1. Which were subsequently integrated as weighted features into the machine learning models.

### 2.5. Model Training and Validation

Five machine learning algorithms were implemented for comparison in this study: Gradient Boosting, Logistic Regression (LR), Random Forest (RF), Support Vector Machine (SVM), and XGBoost. Gradient Boosting sequentially builds weak learners to minimize prediction errors in a stage-wise manner. LR is a conventional linear classifier that estimates the probability of class membership. RF is an ensemble method that constructs multiple decision trees and aggregates their outputs to improve robustness and reduce overfitting. SVM identifies an optimal separating hyperplane in the feature space and is effective for handling complex classification boundaries. XGBoost is an advanced gradient boosting framework that incorporates regularization and optimized tree learning to improve predictive performance. These algorithms were selected to compare linear and non-linear classification strategies and to evaluate both interpretable and high-performance ensemble methods. The final model was selected based on the highest predictive performance on the validation set.

The model was trained and validated using the collected clinical multicenter data. The dataset was partitioned into a training set (70%), validation set (15%), and independent test set (15%) through stratified random sampling to preserve consistency in data distribution. Five classical machine learning algorithms—random forest, XGBoost, logistic regression, support vector machine (SVM), and gradient boosting—were employed for model development, with cross-validation and iterative refinement conducted over 10,000 cycles.

When the validation accuracy decreases by more than 20%, the self-repair mechanism is automatically activated. The corresponding corrective actions are as follows: for the Random Forest model, the number of trees and maximum tree depth are increased; for Gradient Boosting and XGBoost models, the learning rate is reduced; for Logistic Regression, the number of training iterations is increased; and for SVM, the regularization parameter C is increased.

### 2.6. Statistical Analysis

**Figure 2:**
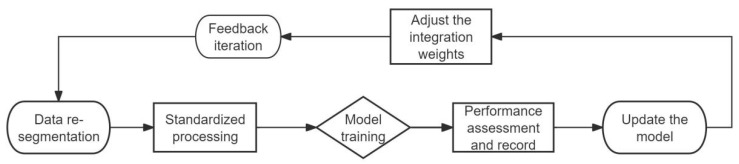
Schematic Diagram of Model Training Process.

Model performance was evaluated using standard metrics, including accuracy, precision, recall, F1-score and confusion matrix (Figure 2). Class-specific accuracy was computed for each diagnostic category to assess per-class performance. The area under the receiver operating characteristic curve (AUC) was also calculated to provide a comprehensive assessment of the model’s classification capability. Confidence intervals for all performance metrics were estimated using the Bootstrap method with 1000 resampling iterations to ensure robustness and reliability of the results.

## 3. Results

### 3.1. Demographic and Clinical Characteristics

This study collected vision health data from 1436 children and adolescents aged 6 to 12 years. The dataset includes multidimensional valid records, such as visual acuity measurements, refractive parameters, and ocular biometric indicators. Following standard machine learning practices, the full dataset was partitioned into a training set (70%), a validation set (15%), and a test set (15%). Detailed data summaries are provided in Table 5.

### 3.2. Model Performance

#### 3.2.1. Training Time of the Five Models

**Figure 3:**
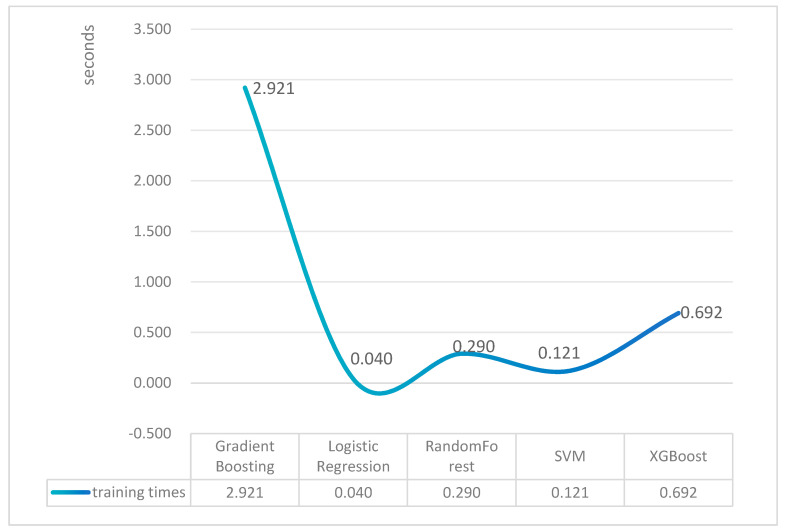
The training times of five different machine learning algorithm models. The training times of the five models—Random Forest, XGBoost, Logistic Regression, SVM, and Gradient Boosting.

The training times of the five machine learning models are shown in Figure 3. Among the compared algorithms, Logistic Regression required the shortest training time (0.040 s), followed by SVM (0.121 s), Random Forest (0.290 s), and XGBoost (0.692 s). Gradient Boosting required the longest training time (2.921 s). These results indicate notable differences in computational efficiency across the models.

#### 3.2.2. Overall Classification Performance

On the independent test set, Gradient Boosting achieved the best overall performance, with an accuracy of 98.67%, an F1 score of 98.67%, and a mean AUC of 0.957. XGBoost ranked second in overall classification performance, followed by Random Forest, Logistic Regression and SVM. These findings were consistent across the different evaluation metrics. The detailed data are presented in Table 6.

#### 3.2.3. ROC Curve Analysis

**Figure 4:**
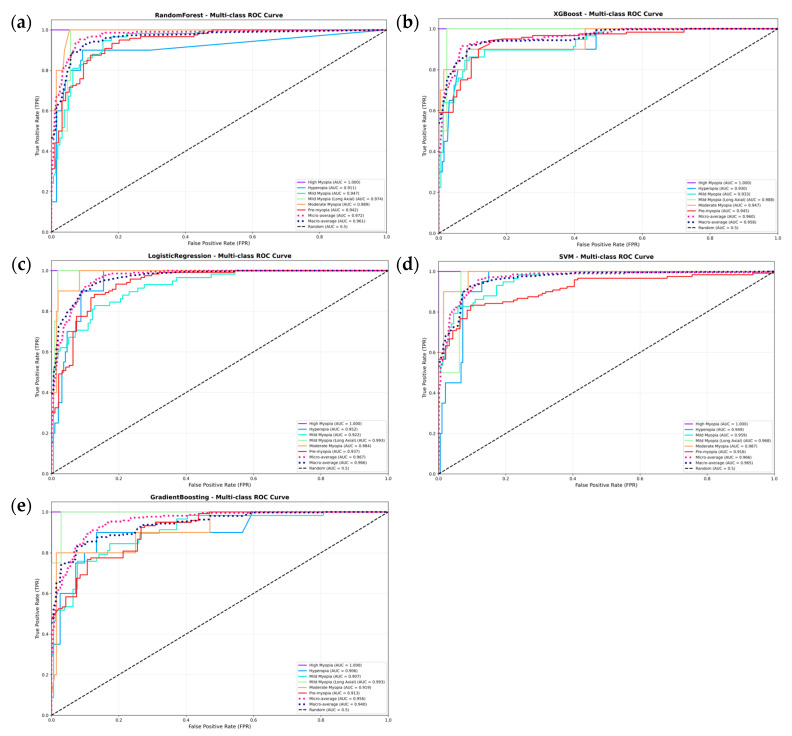
ROC curves of predictive models trained with five distinct machine learning methodologies. The AUC values of the five models for diagnosing various refractive states all exceeded 0.900. (**a**) Random Forest multi-class ROC curves; (**b**) XGBoost multi-class ROC curves; (**c**) Logistic Regression multi-class ROC curves; (**d**) SVM multi-class ROC curves; (**e**) Gradient Boosting multi-class ROC curves.

Figure 4 presents the ROC curves of the five machine learning models on the independent test set. All models achieved favorable discriminative performance, with AUC values exceeding 0.900 across the five refractive categories. The Random Forest model showed strong ROC performance across the classes, while the other models also maintained high discriminatory ability. The ROC curves remained well above the diagonal reference line, indicating performance better than random classification.

#### 3.2.4. Learning Curves and Model Generalization

**Figure 5:**
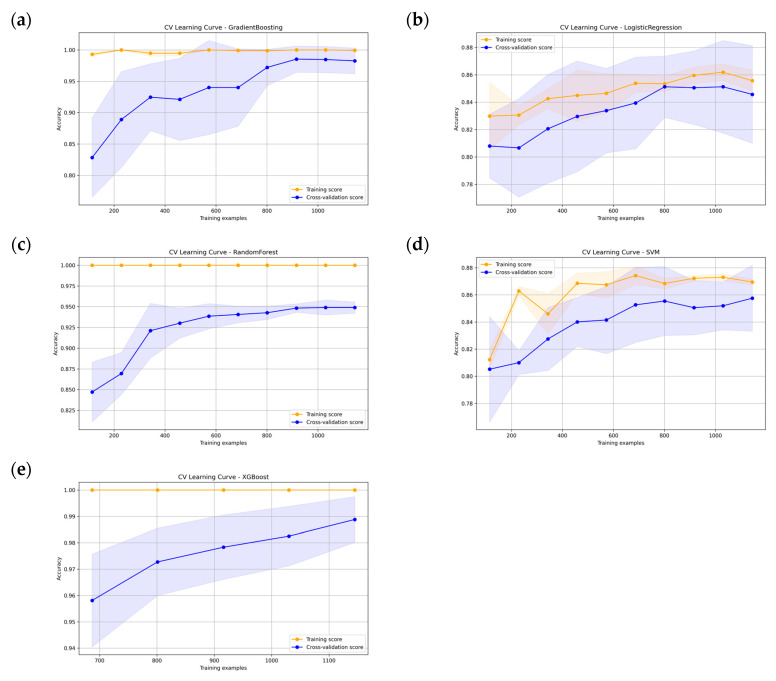
Learning curves of predictive models trained with five distinct machine learning methodologies. (**a**) Gradient Boosting; (**b**) Logistic Regression; (**c**) Random Forest; (**d**) SVM; and (**e**) XGBoost. The learning curves depict the training and cross-validation accuracy as a function of the number of training examples. The shaded areas indicate the standard deviation across cross-validation folds.

Figure 5 presents the learning curves of the five models. Gradient Boosting, XGBoost, and Random Forest all showed high training accuracy, with cross-validation curves remaining below the training curves across sample sizes. Logistic Regression and SVM showed lower accuracy values overall, but the difference between training and cross-validation performance was relatively small. The shaded regions around the cross-validation curves indicate variability across folds. Collectively, the curves suggest that the ensemble methods fitted the training data more strongly, whereas Logistic Regression and SVM exhibited more balanced training and validation performance.

#### 3.2.5. Confusion Matrix Analysis

**Figure 6:**
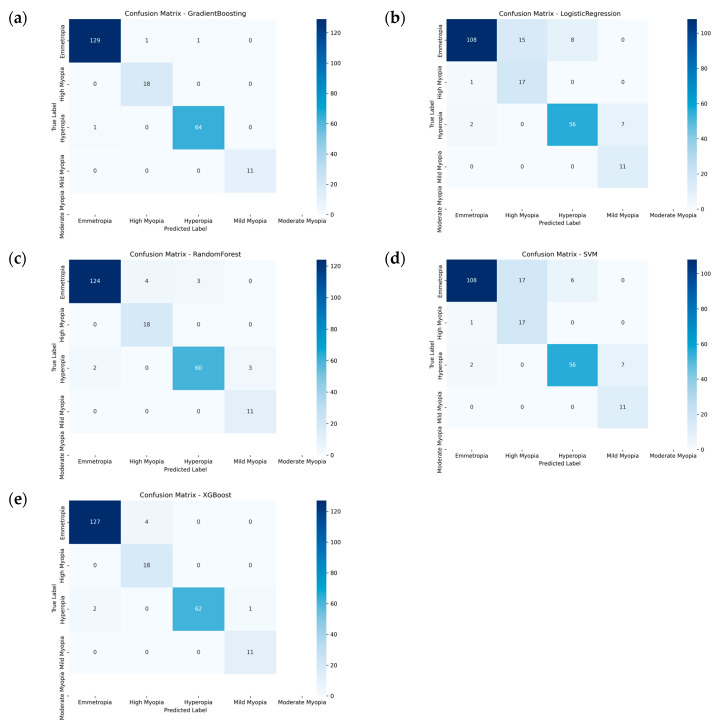
Confusion matrices of the five machine learning models for multiclass refractive status classification. (**a**) Gradient Boosting; (**b**) Logistic Regression; (**c**) Random Forest; (**d**) SVM; and (**e**) XGBoost. Rows indicate the true labels, and columns indicate the predicted labels. The diagonal elements represent correct classifications, whereas off-diagonal elements indicate misclassifications.

Figure 6 illustrates the confusion matrices of the five machine learning models. All models showed a clear diagonal pattern, indicating satisfactory multiclass classification performance. Random Forest and XGBoost achieved the most favorable class separation, whereas Logistic Regression and SVM demonstrated relatively more frequent confusion among adjacent refractive categories. Notably, misclassification patterns were predominantly observed between clinically similar states, rather than between distant refractive classes, suggesting that the models captured the graded nature of refractive status classification.

## 4. Discussion

Myopia is a prevalent refractive disorder that typically emerges during childhood, with its prevalence increasing progressively with age. Numerous studies have reported that the myopia rate among Chinese students is approximately 42%, rising to as high as 95.5% in certain regions [17]. It has become a major public health concern in China. According to national survey data, the overall myopia prevalence among children and adolescents aged 6–18 reached 52.7% in 2020, affecting an estimated 100 million individuals. In the absence of effective intervention strategies, this rate may rise further in the coming years [18,19,20]. Therefore, early identification, risk stratification, and timely intervention are essential for reducing the long-term burden of myopia.

In clinical and epidemiological studies, myopia is most commonly defined by spherical equivalent (SE), although the cutoff values vary across studies. The most frequently used thresholds include −0.50 D, −0.75 D, and −1.00 D or worse [21,22,23]. Some studies adopt a broader criterion, defining myopia as SE exceeding −0.25 D [24]. Likewise, there is no universally accepted definition of high myopia. Various studies have defined high myopia using SE thresholds of −5.00 D, −6.00 D, or −8.00 D or worse, or by axial length criteria such as >25.5 mm, >26.0 mm, or >26.5 mm [25,26,27]. In this study, we adopted the non-cycloplegic definition of myopia as SE ≤ −0.75 D, consistent with current population-based screening practice and methodological trends in large-scale pediatric refractive surveys. This choice is also more suitable for real-world screening environments in which cycloplegic examination is not always feasible.

Axial length (AL) played an important complementary role in the present model. Unlike refractive measurements, which are affected by accommodation and cooperation during examination, AL is an objective biometric parameter that reflects ocular structural elongation. In children and adolescents, AL increases progressively with growth and is closely associated with the onset and progression of myopia [28,29,30,31]. In our model, AL was not used as the sole diagnostic criterion. Instead, it served as an auxiliary structural indicator to refine classification, especially in borderline cases such as emmetropia and pre-myopia. By comparing AL with age-matched reference ranges, the model could identify children whose refractive status appeared near normal but whose ocular structure suggested an elevated risk of future myopia. This design allowed the model to move beyond a purely refraction-based approach and incorporate more meaningful structural information.

The axial length-to-corneal radius ratio (AL/CR) provided another important layer of information. AL/CR is a combined biometric index that reflects the relationship between axial elongation and corneal curvature, thereby representing the overall refractive architecture of the eye. Previous studies have shown that AL/CR is strongly correlated with refractive error and may outperform AL alone in myopia detection [32,33]. In clinical practice, AL/CR is especially useful for distinguishing axial myopia from refractive myopia because it integrates both ocular elongation and corneal morphology. For example, a child with a relatively long axial length may not necessarily be myopic if the corneal curvature is correspondingly flatter, whereas another child with a steeper cornea may already demonstrate myopia despite having an axial length within the age-expected range. Therefore, AL/CR offers a more comprehensive and clinically informative biomarker than AL alone. In our model, AL/CR was incorporated both into the rule-based logic and into the machine learning feature set, enabling the algorithm to capture clinically relevant structural differences that are not fully reflected by SE alone.

Importantly, the present model was designed as a non-cycloplegic screening-support tool rather than a replacement for comprehensive ophthalmic examination. Although cycloplegic refraction remains the gold standard for precise diagnosis, routine cycloplegia for every child is often impractical in large-scale screening programs because it is time-consuming, requires additional clinical workflow, and may reduce cooperation from children and parents. In this context, our model provides a pragmatic and scalable alternative for preliminary screening and risk stratification. By combining refractive parameters with objective biometric indicators such as AL and AL/CR, the model can improve diagnostic accuracy under non-cycloplegic conditions and reduce the limitations of relying on a single refraction-based measurement. This feature makes the model particularly suitable for school-based screening and primary care settings, where rapid assessment of large numbers of children is required. In our study, to achieve strong diagnostic performance, with the best-performing model (Gradient Boosting) attaining an accuracy of 98.67%, an F1 score of 98.67%, and a mean AUC of 0.957 on the independent test set.

In addition to its screening utility, the model also has important implications for clinical decision support. The rule-enhanced AI framework integrates explicit diagnostic thresholds derived from clinical guidelines with data-driven machine learning algorithms, allowing the model to retain clinical interpretability while achieving strong predictive performance. In practice, this means that the model can assist clinicians in identifying children at risk of myopia or progression and can support more individualized recommendations for follow-up and intervention. Because the system incorporates both rule-based reasoning and machine learning, it is more transparent than many conventional black-box models and may therefore be more acceptable to clinicians in routine practice.

From a public health perspective, the ability of the model to be deployed locally is another major advantage. All data processing can be completed within a secure internal server environment, which avoids the need to transfer sensitive patient information to external cloud platforms. This design supports privacy protection and is consistent with current medical data security requirements. More importantly, local deployment makes the model more feasible for use in large-scale school screening programs and community-based health systems, where high throughput, standardized evaluation, and data security are all critical.

The model is particularly well aligned with the needs of myopia prevention and control in China. Given the extremely large number of children requiring screening, it is unrealistic to perform cycloplegic refraction in every case. A non-cycloplegic, multi-parameter AI model can therefore serve as an efficient first-line auxiliary tool to identify children who require closer monitoring or referral. This approach is consistent with the current public health strategy in China, which emphasizes early prevention, early detection, and early intervention. By supporting rapid screening in a standardized and privacy-preserving manner, the model may help improve the efficiency of myopia control programs and reduce the burden of high myopia in the future.

This study has several strengths. First, it adopts a rule-guided AI framework that translates clinical guidelines into executable logic, thereby improving interpretability and clinical consistency. Second, the model combines refractive and biometric parameters to capture both functional and structural aspects of myopia, which is particularly valuable in non-cycloplegic screening scenarios. Third, the model supports local deployment, making it suitable for use in settings where data security and operational efficiency are essential. Finally, the model demonstrates strong diagnostic performance across multiple machine learning algorithms, suggesting good potential for real-world application.

However, this study still has some limitations. While the results of internal cross-validation and independent test sets indicate strong diagnostic performance, a significant limitation of this study is its reliance on retrospective data from a single clinical center. This may limit the immediate generalizability of the model in a broader population with diverse ethnic backgrounds or socioeconomic statuses. To address this issue, our future research plans include a comprehensive external validation phase. We intend to deploy the model in a prospective, multicenter study covering various primary healthcare institutions and school screening programs across different geographic regions of China. Future external validation will not only test the model’s robustness on different hardware devices, such as various automated refractometers and biometers, but will also allow for continuous refinement of the rule-enhanced algorithm in a large-scale, real-world clinical setting. Furthermore, this model is designed for cross-sectional diagnosis, not for tracking or predicting the progression of myopia over time. Incorporating longitudinal follow-up data, as well as environmental and genetic variables, is crucial for developing more comprehensive predictive tools in future research. Finally, while our rule-enhanced multi-parameter AI framework offers a conceptually different approach compared to most existing myopia AI tools—which typically rely on a single input pattern—it has not yet been rigorously compared directly with other published systems. This reflects the broader challenge of benchmarking AI models across studies with varying dataset compositions, diagnostic definitions, and evaluation methods. Future external validation research should fill this gap through standardized comparative evaluations.

## 5. Conclusions

In conclusion, we developed a rule-enhanced, multi-parameter AI diagnostic model for pediatric myopia that is interpretable, privacy-preserving, and suitable for non-cycloplegic screening. By integrating SE, AL, AL/CR, and other ocular parameters, the model provides a practical solution for large-scale myopia screening and risk stratification, particularly in China where cycloplegic examination for every child is unrealistic. This approach may support earlier identification of at-risk children and contribute to more effective myopia prevention and control strategies.

## Figures and Tables

**Table 4:** Diagnostic Classification of Refractive Status.

Refractive Status	Definition
Hyperopia	Following cycloplegic refraction (dilated eye examination), the spherical equivalent refractive power exceeds the upper limit of hyperopic reserve for age-matched children.
Pre-myopia group	−0.50 D ≤ spherical equivalent (SE) ≤ +0.75 D, in conjunction with baseline refractive error, age, and other quantifiable risk factors, indicates a high likelihood of future myopia development.
Low myopia group	−0.50 D < SE ≤ −3.00 D;
Moderate myopia group	−3.00 D < SE ≤ −6.00 D;
High myopia group	SE > −6.00 D

**Table 5:** Demographic and Clinical Characteristics of Participants in the Developmental and Test Datasets.

	Developmental Dataset		Test Set
	Training Set	Tuning Set	
Number of participants	1006	216	214
Gender (Boys %)	49.7%	43.5%	48.6%
Age (years)	8.48 ± 1.83	8.51 ± 1.84	8.83 ± 1.85
Vision (logMAR)	0.68 ± 0.3	0.71 ± 0.31	0.69 ± 0.3
SE (D)	−0.66 ± 1.48	−0.82 ± 2.13	−0.73 ± 1.58
AL (mm)	23.56 ± 1.53	23.59 ± 1.78	23.60 ± 1.05
AL/CR	3.05 ± 0.84	3.03 ± 0.14	3.05 ± 0.12
Hyperopia (%)	108 (10.74%)	30 (13.89%)	20 (9.35%)
Pre-myopia (%)	530 (52.68%)	108 (50%)	120 (56.07%)
Mild myopia (%)	312 (31.01%)	58 (26.85%)	62 (28.97%)
Moderate myopia (%)	49 (4.87%)	16 (7.41%)	10 (4.67%)
High myopia (%)	7 (0.7%)	4 (1.85%)	2 (0.93%)

Data are presented as mean ± standard deviation, number (percentage), or as otherwise indicated. Vision was measured and is presented in logMAR units. Spherical Equivalent (SE) was calculated as sphere + ½cylinder. AL/CR ratio: Axial Length to Corneal Radius of curvature ratio. The Developmental dataset was used for model development, internally split into a Training set for model building and a Tuning set for hyperparameter optimization. The independent Test set was used for final model evaluation.

**Table 6:** Comparison of model performance on the independent test set (n = 214).

Model	Accuracy (%)	Precision (%)	Recall (%)	F1 Score (%)	Mean AUC (95% CI)
Gradient Boosting	98.67%	98.69%	98.67%	98.67%	0.957 (0.930–0.984)
Logistic Regression XGBoost	85.33%	89.16%	85.33%	86.20%	0.962 (0.936–0.988)
Random Forest	94.67%	95.20%	94.67%	94.77%	0.960 (0.934–0.986)
SVM	85.33%	89.73%	85.33%	86.38%	0.939 (0.907–0.971)
XGBoost	96.89%	97.24%	96.89%	96.96%	0.965 (0.940–0.990)

All models were trained with 10,000 iterations. Performance metrics were evaluated on the independent test set (n = 214). 95% confidence intervals (CIs) were calculated using the Wilson score method for the Hanley-McNeil approximation for AUC. The best-performing model is highlighted in bold.

## Data Availability

All data utilized in this study were collected from the Pediatric Ophthalmology and Strabismus Refractive Error Clinic at the Second Affiliated Hospital of Dalian Medical University. To ensure patient privacy, the datasets are not publicly accessible. Requests for access to the data should be directed to the corresponding author. The code for this project is available for download at https://github.com/siqizhang989/Myopia-diagnosis.git (accessed on 1 February 2026).
